# Supporting One’s Own? Grandparents’ Help to Grandchildren Who Live With Other Unrelated Children

**DOI:** 10.1093/geroni/igab046.1904

**Published:** 2021-12-17

**Authors:** Teresa Cooney

**Affiliations:** University of Colorado Denver, Denver, Colorado, United States

## Abstract

The structures of young families today are becoming increasingly complex, which may impact grandparents’ involvement. I examine whether grandparents’ support to adult children’s households differs for those with biological grandchildren only, versus households with both biological and non-biological (step, unrelated) grandchildren. The resource dilution hypothesis and sociobiology theory suggest that grandparents will be less supportive of grandchildren when other unrelated children co-reside in their households. Grandparents (mean age 62.23) in the Add Health Parent Study (2015-2017) reported on instrumental and financial help given to each of their adult children's families in the past year. These data were merged with information from their adult children (mean age 36.76) who participated in Add Health Wave V (2016-2018). Adult children’s household structures—biological children only (n=400) or biological + other children (n=51)—were determined using their fertility histories and household rosters. No significant differences were found in the likelihood that grandparents offered any instrumental or financial support to these two household types (controlling for grandparent resources and adult child characteristics). Nor was the level of grandparents’ financial support significantly different for the two groups. However, grandparents gave significantly fewer hours of help to adult children heading households including both biological grandchildren and unrelated children. Grandparents appear less willing to devote time to assisting their grandchildren’s families when their investment is diluted by the presence of unrelated children. Perhaps time with grandchildren is less pleasing or comfortable when unrelated children are present. This same issue does not impact financial giving, which need not involve contact.

